# Virus-like Particles Formed by the Coat Protein of the Single-Stranded RNA Phage PQ465 as a Carrier for Antigen Presentation

**DOI:** 10.3390/molecules30204056

**Published:** 2025-10-11

**Authors:** Egor A. Vasyagin, Eugenia S. Mardanova, Nikolai V. Ravin

**Affiliations:** Institute of Bioengineering, Research Center of Biotechnology of the Russian Academy of Sciences, 119071 Moscow, Russia

**Keywords:** virus-like particle, self-assembly, coat protein, protein display, recombinant vaccine, ssRNA bacteriophage, transient expression, *Nicotiana benthamiana*

## Abstract

Virus-like particles (VLPs) formed as a result of self-assembly of viral capsid proteins are widely used as a platform for antigen presentation in vaccine development. However, since the inclusion of a foreign peptide into the capsid protein can alter its spatial structure and interfere with VLP assembly, such insertions are usually limited to short peptides. In this study, we have demonstrated the potential of capsid protein (CP) of single-stranded RNA phage PQ465 to present long peptides using green fluorescent protein (GFP) as a model. GFP was genetically linked to either the N- or C-terminus of PQ465 CP. Hybrid proteins were expressed in *Escherichia coli* and *Nicotiana benthamiana* plants. Spherical virus-like particles (~35 nm according to transmission electron microscopy) were successfully formed by both N- and C-terminal fusions expressed in *E. coli*, and by plant-produced CP with GFP fused to the C-terminus. ELISA revealed that GFP in VLPs was accessible for specific antibodies suggesting that it is exposed on the surface of PQ465-GFP particles. VLPs carrying GFP were recognized by anti-CP antibodies with less efficiency than VLPs formed by empty CP, which indicates shielding of the CP core in PQ465-GFP particles. Therefore, PQ465 CP can be used as a chimeric VLP platform for the display of relatively large protein antigens, which can operate in bacterial and plant expression systems.

## 1. Introduction

Viruses can infect a wide range of organisms, both prokaryotes and eukaryotes. A distinctive feature of viruses is the ability of their capsid proteins to spontaneously self-assemble to form virus-like particles (VLPs). Various recombinant expression systems can be used to produce VLP-forming proteins, including bacteria, yeasts, plants, and insect and mammalian cells. VLPs are used in drug delivery, diagnostic tests, as well as in vaccine development [1,2]. VLPs are tridimensional shaped structures that can induce both humoral and cellular immune responses [3,4]. VLPs mimic their parental viruses in terms of size, structure, and the ability to induce potent humoral and cellular immune responses [4]. Some VLP-based vaccines were approved for use, including vaccines against human papillomavirus (HPV), hepatitis B virus, hepatitis E virus [5,6,7]. VLP-based vaccine candidates against hepatitis A virus [8], Chikungunya virus [9], Ebola virus [10], influenza A virus [11], Norovirus [9], Norwalk virus, Respiratory syncytia virus, Rotavirus [10], SARS-CoV-2 [12] are at different steps of preclinical and clinical trials, and a huge number of VLP-based vaccines are under development [4,13].

Additionally, VLPs can serve as platforms to display foreign peptide antigens, resulting in the formation of chimeric VLPs. The goal of chimeric VLPs is to enhance the immunogenicity of foreign peptides displayed on the surface. VLPs derived from animal, plant and bacterial viruses have been used as a platform for the development of vaccine candidates against other viral infections [4]. Bacterial viruses, bacteriophages, are one of the most promising platforms for VLP engineering because their capsid proteins can be efficiently and cost effectively produced in bacterial expression systems. The single-stranded RNA (ssRNA) bacteriophages of the family *Fiersviridae* (formerly known as *Leviviridae*) are particularly promising for VLP design. They are small viruses containing linear ssRNA(+) genome about 3.4–4.3 kb in size. Spherical virions are about 28 nm in diameter consisting of about 180 copies of the major coat protein (CP) and one or two species of minor structural proteins. Notably, recombinant expression of a single capsid protein results in the formation of VLPs that are morphologically very similar to native virions. VLPs have been developed from CPs of bacteriophages AP205, MS2, PP7, Qβ, P22 [4]. Bacteriophage AP205 VLPs have been used as carriers for West Nile virus antigens [14] and M2e peptides of influenza A virus [15]. VLPs formed by capsid proteins of bacteriophages MS2 and PP7 were used to present HPV antigens [16,17].

However, there are also problems with using bacteriophage capsid proteins as building blocks for construction of hybrid VLPs. Incorporation of a foreign peptide into the capsid protein changes its spatial structure and can interfere with VLP assembly due to steric limitations. As a rule, the larger the insertion size, the more it changes the structure of the chimeric protein, so the size of the included peptides is usually limited to units to tens of amino acid residues, which does not allow to apply this approach to present full-length peptide antigens or multiple peptide epitopes, which usually elicit a stronger and more diverse immune response than short epitopes.

However, the capabilities of VLPs based on the above-mentioned phages AP205, MS2 and PP7 for display of long polypeptides are relatively limited. Studies on the AP205 VLP platform have demonstrated its capacity for C-terminal and N-terminal fusions of only up to 55 a.a. and 39 a.a., respectively [18]. The assembly of MS2 VLPs is also strictly constrained by insert size, and functional VLPs formed when the inserted sequences were less than 91 a.a. in length [19]. Bacteriophage PP7-based VLP was shown to tolerate the display of up to 124 a.a. long sequences on its surface as C-terminal genetic fusions to the coat protein [20]. Therefore, the search for new VLPs as carriers of longer polypeptides is of great importance for the development of vaccines.

Large-scale metagenome sequencing made it possible to identify new hypothetical bacteriophages that can be used as a source of new VLPs. More than a hundred new hypothetical ssRNA bacteriophages were discovered in just a single study [21]. The capsid proteins of 110 bacteriophages were expressed in *E. coli*, and 80 of them were able to form VLPs [22]. Forty-three novel phage capsid proteins were tested for their ability to form chimeric VLPs containing genetically fused triple repeat of the 23 N-terminal amino acid residues of the influenza A virus M2 protein (M2e). Capsid proteins of ten ssRNA phages were able to form chimeric VLPs, and five of them tolerated both N- and C- terminal insertions [23].

We selected the CP ssRNA phage PQ465 as a promising candidate for vaccine development. Although the sequence of PQ465 CP is only 30% identical to that of well characterized Acinetobacter phage AP205, there are no significant differences in the three-dimensional structures of the two proteins [24]. The spatial location of the CP termini in VLPs is the most important in the context of chimeric fusion proteins. The ends of the AP205 coat protein are exposed on the surface and are therefore suitable for fusions [25]. The VLPs were very stable, and the AP205 coat protein was found to be very hard, tolerating relatively large insertions (several dozen a.a.) at both the N- and C-termini [18].

In this work, we investigated the possibility of obtaining, on the basis of the chimeric capsid protein of the phage PQ465, VLPs carrying longer polypeptides, the size of which is comparable to full-length antigens. As an inserted model polypeptide, we used green fluorescent protein, which has a length of 238 a.a. (26.8 kD).

VLPs can be produced in various expression systems including bacterial, yeast, insect, plant, and mammalian cells. The bacterial expression system, particularly in *Escherichia coli*, is characterized by simplicity, low cost, high speed, and, in addition, when expressing bacteriophage capsid proteins, it provides a high level of synthesis of the target product [23]. Its limitations include the possibility of contamination of the product with endotoxins and the absence of post-translational modifications characteristic of eukaryotes, which can alter the properties of the recombinant protein. The advantage of the plant expression system is the safety of the product, since plants and humans do not have common pathogens, and the use of the transient expression method allows one to obtain the protein of interest in about a week. The use of two platforms for expression of recombinant protein will allow the selection of the optimal system for a particular protein. Testing two platforms for recombinant protein expression will make it possible to select the optimal system for a specific protein preparation.

In this study, we obtained fusion proteins containing GFP genetically linked to the N- or C-termini of the capsid protein of phage PQ465. Recombinant proteins, expressed in *E. coli* and *Nicotiana benthamiana* plants, were able to form VLPs displaying functionally active GFP thus demonstrating that the PQ465 capsid protein can be used as a carrier of relatively long antigens in vaccine development.

## 2. Results

### 2.1. Expression in E. coli and Purification of Recombinant Fusion Proteins Consisting of the Coat Protein of Phage PQ465 Linked to GFP

**Figure 1:**
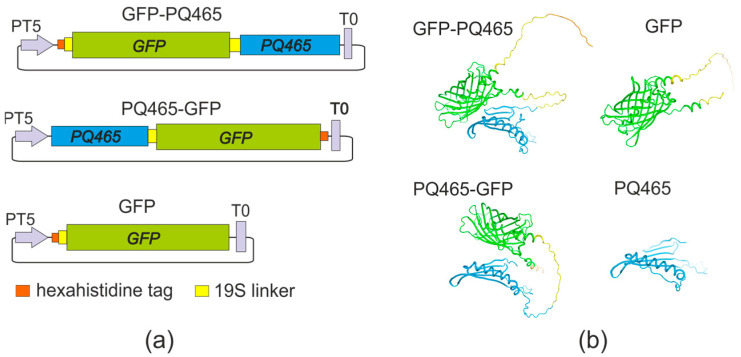
Structures of recombinant proteins. (**a**) Schematic representation of the expression constructs. PT5, bacteriophage T5 promoter; T0, lambda T0 terminator; PQ465, coat protein of bacteriophage PQ465; GFP, green fluorescent protein. (**b**) Predicted structures of recombinant proteins.

We have designed vectors for the expression of PQ465 capsid protein with GFP at N- or C-termini in *E. coli* (Figure 1). GFP was linked to the capsid protein at the N- or C-terminus via a flexible 19S glycine-serine linker to facilitate proper folding of the fusion protein, VLP assembly, and ensure efficient exposure of the antigen on the VLP surface.

**Figure 2:**
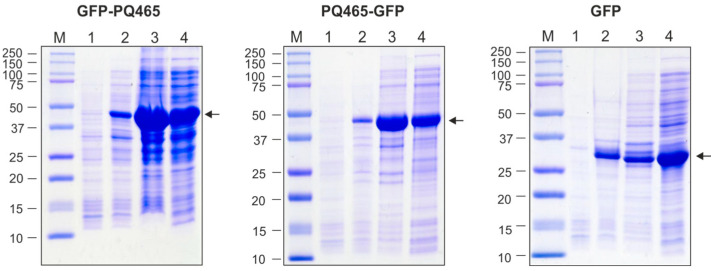
Production of recombinant proteins in *E. coli*. M, molecular weight marker (kD); protein samples from *E. coli* cells before induction (total protein, lane 1), upon induction (total protein, lane 2; insoluble fraction, lane 3; soluble fraction, lane 4). Arrows indicate the positions of target proteins.

Recombinant proteins GFP-PQ465 and PQ465-GFP, as well as GFP alone, were expressed in *E. coli* at high levels. Recombinant proteins comprising PQ465 capsid and GFP were observed in both soluble and insoluble fraction, while GFP was predominantly soluble (Figure 2).

**Figure 3:**
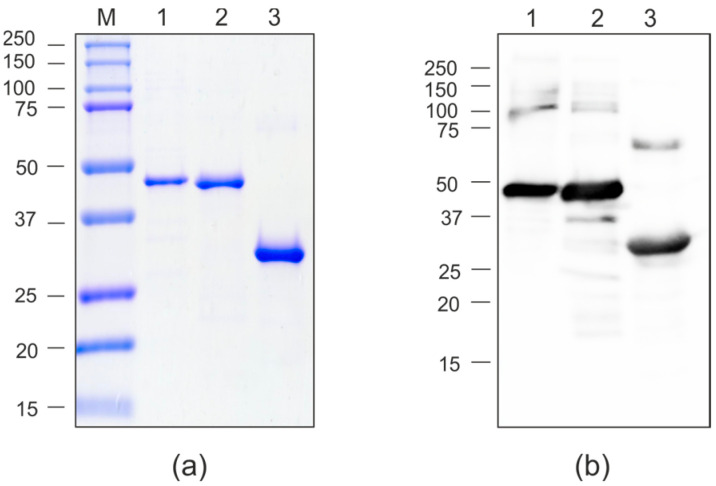
Purification of the recombinant proteins expressed in *E. coli*. (**a**), Coomassie brilliant blue-stained SDS-PAGE gel of purified proteins; (**b**), Western blot analysis with antibodies against GFP. M, molecular weight marker (kD); 1, GFP-PQ465; 2, PQ465-GFP; 3, GFP.

Clarified lysates of soluble fractions of PQ465-GFP, GFP-PQ465 and GFP were used for protein purification. VLPs formed by PQ465-GFP and GFP-PQ465 were purified by precipitation of the cell lysate with saturated ammonium sulfate overnight at 4 °C. Purification of the GFP was performed using metal-affinity chromatography. All proteins were purified to homogeneity and were recognized by anti-GFP antibodies (Figure 3). Expression and purification of the empty PQ465 capsid protein were described previously [26].

### 2.2. VLPs Formed by PQ465 CP-Based Proteins Produced in E. coli

**Figure 4:**
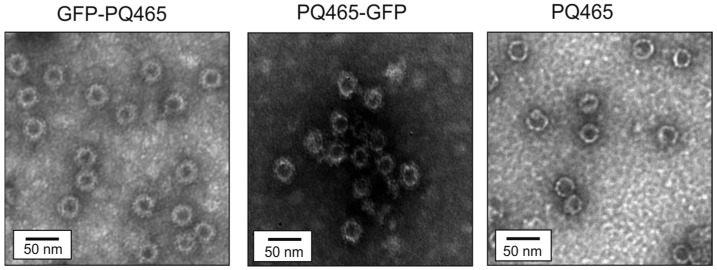
Analysis of VLPs using transmission electron microscopy.

Assembly of recombinant proteins PQ465-GFP and GFP-PQ465, as well as control PQ465 CP into VLPs was analyzed by dynamic light scattering (DLS) and transmission electron microscopy (TEM). According to DLS measurements, the samples contained particulate structures about 40–45 nm in size (Table 1). Examination of the PQ465-GFP and GFP-PQ465 samples by TEM revealed VLPs with size of about 35 nm, while empty PQ465 CP without insertions formed slightly smaller particles of about 30 nm in size (Figure 4).

### 2.3. Exposure of GFP on the Surface of Chimeric VLPs

**Figure 5:**
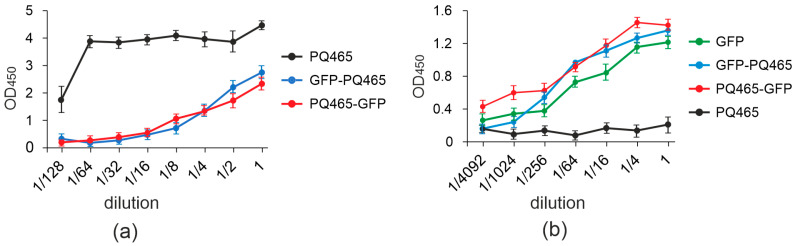
Antigenic properties of recombinant proteins. The dilutions of recombinant proteins GFP-PQ465, PQ465-GFP, PQ465, GFP were applied to ELISA plates, which were then probed with antibodies against PQ465 CP (**a**) or GFP (**b**). Mean values and error bars representing standard deviation (measurements from three wells) are shown.

We showed that fusion of GFP to the N- or C-terminus of the phage PQ465 capsid protein does not affect VLP formation. However, not only the ability to form VLPs is important, but also the spatial arrangement of attached antigens inthe particles. It is desirable that the antigen be located on the surface of the particle, in which case it should be recognized by specific antibodies with approximately the same efficiency as the “free” protein. The antigenic properties of the obtained VLPs were assessed using ELISA with specific antibodies against GFP and empty PQ465 capsid protein (Figure 5).

The obtained results revealed that VLPs formed by both fusions, PQ465-GFP and GFP-PQ465, and “free” GFP exhibited similar binding to anti-GFP antibodies. At the same time, binding of antibodies against the PQ465 capsid to the “empty” PQ465 VLPs occurred more efficiently than to the fusions, indicating that the attached GFP reduces the availability of CP for the antibodies. Thus, the obtained results indicate that GFP is exposed on the surface of recombinant VLPs and effectively masks the carrier protein from binding to specific antibodies, which plays an important role in the overall vaccine design.

### 2.4. Expression of Fusion Proteins in N. benthamiana Plants

**Figure 6:**
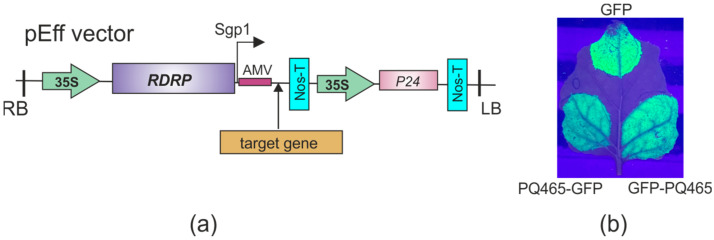
Expression of recombinant proteins in *N. benthamiana* plants. (**a**) Scheme of the expression vector pEff. 35S, promoter of the cauliflower mosaic virus RNA; RDRP, RNA-dependent RNA polymerase gene; Sgp1, promoter of the first subgenomic RNA of PVX; AMV, translational enhancer from alfalfa mosaic virus; Nos-T, terminator of the *A. tumefaciens nos* gene; P24, silencing suppressor from grapevine leafroll-associated virus-2; LB and RB, the left and right borders of T-DNA region. (**b**) Visualization of GFP fluorescence in *N. benthamiana* leaves infiltrated with expression vectors.

**Figure 7:**
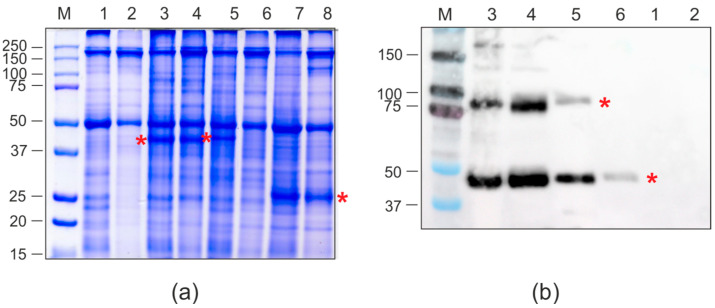
Production of recombinant proteins in *N. benthamiana* plants. SDS-PAGE (**a**) and Western blotting with antibodies against hexahistidine tag (**b**). M, molecular weight marker (kD); Lanes: 1, total proteins isolated from non-infiltrated leaf; 2, soluble proteins isolated from non-infiltrated leaf; 3, total proteins isolated from leaf infiltrated with pEff-PQ465-GFP; 4, soluble proteins isolated from leaf infiltrated with pEff-PQ465-GFP; 5, total proteins isolated from leaf infiltrated with pEff-GFP-PQ465; 6, soluble proteins isolated from leaf infiltrated with pEff-GFP-PQ465; 7, total proteins isolated from leaf infiltrated with pEff-GFP; 8, soluble proteins isolated from leaf infiltrated with pEff-GFP. Positions of recombinant proteins are shown by asterisks.

Having successfully demonstrated the expression of fusion proteins and VLP assembly in bacterial systems, we have implemented this technology in plant-based expression platform. PQ465 CP, GFP, and CP-GFP fusions were expressed in *N. benthamiana* using the pEff viral vector [27]. *N. benthamiana* leaves were infiltrated with agrobacteria carrying recombinant vectors and GFP fluorescence was monitored by UV irradiation (Figure 6). We observed GFP fluorescence for both fusions, which was slightly lower in intensity compared to the control GFP. The protein samples were extracted from the leaves and analyzed using SDS-PAGE (Figure 7a) and Western blotting (Figure 7b). The results showed that PQ465-GFP and GFP-PQ465 proteins were efficiently expressed in *N. benthamiana* plants accounting for about 20% and 10% of the total protein, respectively. Analysis of total and soluble protein fractions showed that PQ465-GFP was present in the soluble fraction, whereas GFP-PQ465 protein was predominantly insoluble, but we attempted to purify it from the soluble fraction. Rather unexpectedly, in the case of PQ465 CP expression, we did not observe the appearance of the target protein band on SDS-PAGE and were unable to purify the empty capsid protein.

**Figure 8:**
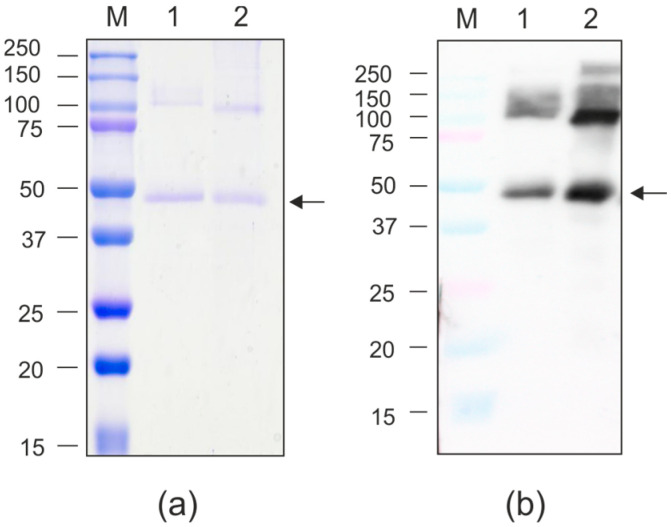
Purification of the recombinant proteins expressed in *N. benthamiana*. (**a**) Coomassie brilliant blue-stained SDS-PAGE gel; (**b**) Western blot analysis with antibodies against GFP. M, molecular weight marker (kD); 1, GFP-PQ465; 2, PQ465-GFP. Arrows indicate the positions of monomers of recombinant proteins.

For protein purification, agroinfiltrated leaves were taken on the fourth day after infiltration. Recombinant proteins were purified using metal-affinity chromatography under non-denaturing conditions. Purified proteins were analyzed by SDS-PAGE and Western blotting (Figure 8). The yield of purified PQ465-GFP reached approximately 100 μg per gram of fresh leaf biomass. In contrast, GFP-PQ465 showed 2.5 to 5-fold lower yields owing to its only partial solubility. Both PQ465-GFP and GFP-PQ465 fusion proteins were specifically revealed in Western blotting with the antibodies against GFP. In addition to the bands corresponding in molecular weight to the monomers of the hybrid proteins, larger bands were also observed, approximately corresponding to the position of the dimers.

**Figure 9:**
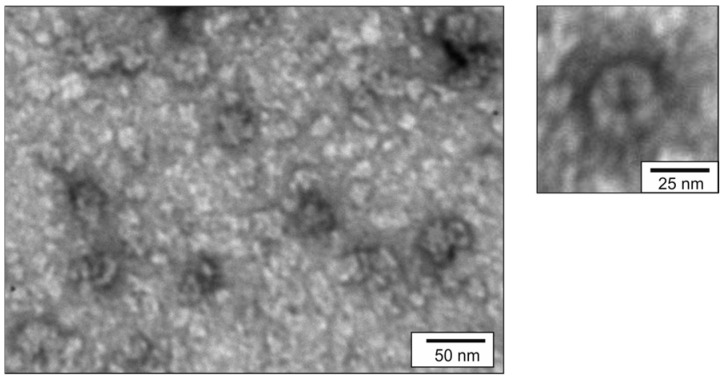
Analysis of VLPs formed by PQ465-GFP produced in *N. benthamiana* using transmission electron microscopy.

Assembly of plant-produced recombinant proteins based on phage PQ465 capsid into spherical VLPs was analyzed by DLS and TEM. VLPs were observed in case of the C-terminal GFP fusion PQ465-GFP (Figure 9), while no particulate structures were detected in GFP-PQ465 samples. The sizes of PQ465-GFP VLPs according to DLS and TEM methods were 41.2 ± 2.5 nm and 32.7 ± 5.2 nm, respectively. These values were in close agreement with the diameters VLPs measured for PQ465-GFP produced in *E. coli*, confirming host-independent assembly of particles with a defined structure.

## 3. Discussion

The variety of VLPs can be engineered to link multivalent antigen structures in vaccine development studies and other applications [3]. VLP are capable of presenting peptide epitopes at high density and in a specific conformation and stimulate both the innate and adaptive immune systems [28]. Due to its advantages, VLPs has already taken its place in the vaccine market, and a number of VLP-based vaccines are now in various phases of clinical trials [1,3,9,29,30].

VLPs can act as vaccines against the parental virus and as carriers for the efficient presentation of foreign antigens [31]. To display foreign antigens on the VLP, genetic fusion techniques or chemical coupling methods can be used. Genetic fusion of a target peptide with VLP-forming carrier protein is a simple method that ensures the presence of the peptide within the particle. However, spatial structure of the insert, its length, isoelectric point, etc., could affect particle formation that is influenced by intermolecular chemical bonds and steric hindrance [32,33,34]. Thus, the correct assembly of VLPs is highly unpredictable. Introduction of a foreign peptide may result in the formation of heterogeneous VLPs, and these peptides may not be optimally displayed, which may lead to a limited immune response to them. As a general rule, the larger the size of the inserted antigen relative to the carrier protein itself, the more it can influence particle assembly due to spatial constraints [35]. However, the ability of the carrier to incorporate extended polypeptides is important for vaccine development, since the integration of full-length antigens or combinations of peptide antigens allows generating a more powerful and broader immune response than could be achieved with short epitopes.

In the current study, we employed capsid protein of ssRNA phage PQ465 (126 a.a.) as a versatile VLP scaffold. The structural simplicity of ssRNA phages of the family *Fiersviridae* provides an ideal platform for rapid vaccine development [36]. This is evidenced by clinical-stage VLP vaccines against HPV (phage MS2), malaria (phage MS2), and influenza (phages Qβ and AP205) [16,18,37,38,39].

In our previous study PQ465 CP was engineered to display four copies of the conserved influenza M2e peptide (92 a.a.) at either its N- or C-terminus. Both fusion proteins formed spherical VLPs ~30 nm in size when expresses in *E. coli*. Mice immunized with C-terminal PQ465-M2e fusion VLPs developed robust M2e-specific IgG responses and survived lethal influenza challenge, whereas N-terminal fusion demonstrated reduced immunogenicity [26]. In this work, we investigated the possibility of using PQ465 CP as a carrier of longer polypeptides, the size of which is comparable to full-length antigens. As a model protein we used GFP, the size of which (238 a.a.) is nearly twice the length of PQ465 capsid protein itself. To ensure free folding of GFP relative to the PQ465 capsid protein, a flexible 19 a.a. long glycine-serine linker was inserted between them.

At the first stage we used a bacterial expression system (*E. coli*) due to its advantages including rapid biomass accumulation and protein production, elaborated simple methods of recombinant protein purification, and demonstrated success with other single-stranded RNA phage CPs [23]. We obtained VLPs formed by CP PQ465 fused to GFP at either the N or C terminus. The hybrid VLPs exhibited characteristic green fluorescence, indicating that the native GFP folding was retained. The observed size of VLPs carrying GFP was close to the size of particles formed by unmodified PQ465 CP, although the fusion protein is more than twice longer. Electron microscopy analysis revealed core–shell architecture of the fusion VLPs, presumably representing a dense CP-formed core and a diffuse poorly visible GFP surface layer.

The spatial positioning of antigens on VLPs critically determines their immunogenic potential. ELISA analysis showed that VLPs carrying GFP (both N- and C-terminal fusions) exhibited equivalent antibody binding affinity as free GFP, indicating a surface localization of GFP on the particles. This is of key importance for vaccine development, as the presented antigen must be effectively recognized by the immune system. Moreover, anti-CP antibody assays revealed significant carrier protein shielding in VLPs formed by GFP fusions. This is also an important beneficial property of the VLP platform, as such shielding allows the immune response to be directed to the embedded antigen rather than to the carrier, which can therefore be reused without triggering a pre-existing immune response.

Having successfully demonstrated bacterial expression of the target proteins, we expanded our production platform to plants. We employed the self-replicating vector pEff, based on the genetic elements of potato virus X (PVX), which has been widely used to express different proteins in plants [40,41,42,43,44,45]. Both PQ465-GFP and GFP-PQ465 fusion proteins were successfully expressed in plants and exhibited characteristic GFP fluorescence. The N-terminal GFP fusion (GFP-PQ465) primarily accumulated in the insoluble fraction, though small amounts could be purified from the soluble fraction. However, no VLPs were detected, suggesting the N-terminal GFP insertion disrupts proper VLP assembly in plant cells. In contrast, the C-terminal fusion (PQ465-GFP) remained soluble and assembled into VLPs structurally indistinguishable from those produced in *E. coli*, confirming the C-terminus as the optimal site for foreign peptide insertion. Solubility analysis using Swiss-Model predicted higher solubility for the PQ465-GFP (solvation index −1.74) fusion compared to GFP-PQ465 (solvation index −3.42), while GFP alone showed the highest solubility (solvation index −0.87). This difference likely became more pronounced in the plant cells, explaining why PQ465-GFP predominantly accumulated in the soluble fraction and efficiently formed VLPs, whereas GFP-PQ465 largely aggregated into insoluble inclusions and failed to assemble. Nevertheless, the phage PQ465 capsid protein in the C-terminal antigen display format may become a versatile scaffold for plant-produced VLP vaccines.

Several VLP platforms have been shown to tolerate relatively large insertions using GFP as a model. For example, GFP has been fused to hepatitis B virus core antigen [46], hepatitis E virus capsid protein [44], and structural protein VP2 of parvovirus B19 [47] while maintaining proper particle assembly. However, previously reported VLPs based on ssRNA bacteriophages were limited only to small inserts. In particular, the capsid protein of phage AP205 was able to tolerate epitopes up to 55 a.a. in length without losing the ability to assemble in the case of C-terminal fusions, whereas in the case of N-terminal fusion this limit was about 39 a.a. [18]. The RBD domain of SARS-CoV-2 (72 a.a.) was genetically fused to the C-terminus of dimerized capsid proteins of phage AP205. The fused VLPs were expressed in *E. coli*, which resulted in insoluble aggregates. These aggregates were denatured in 8 M urea followed by refolding, which reconstituted VLP formation as confirmed by electron microscopy [48]. Fusion of a longer peptide, a 111 a.a. sequence of the virus-neutralizing domain III (DIII) of the West Nile virus glycoprotein E, to the C-terminus of the AP205 coat protein affected the assembly of chimeric VLPs [49]. However, when this peptide was added to CP via amber or opal termination codons, mosaic AP205-DIII VLPs were generated by cultivation in amber- or opal-suppressing *E. coli* strains [49]. The capacity of MS2 VLPs to accommodate insertions of various sizes was investigated using a “single-chain dimer” approach [19]. The findings revealed that successful assembly of functional MS2 VLPs occurred only when the inserted sequences were shorter than 91 a.a., while fusion with GFP was insoluble [19]. HPV L2 peptides (20–31, 17–31, 69–86 a.a.) were separately linked to MS2 VLPs [16]. The capacity of the phage PP7 display platform is somewhat higher, as recombinant VLPs have been shown to tolerate 124 a.a. long sequences in case of fusion to the C-terminus of the coat protein [20].

Overall, to the best of our knowledge, VLPs formed by the capsid protein of phage PQ465 have the highest tolerance to foreign inserts among ssRNA phages. A particular advantage of the PQ465-based platform is its capacity to accommodate large insertions at both the N- and C-termini without compromising VLP formation or surface localization of the insert. Furthermore, demonstrating its robust production potential, the PQ465 capsid protein fused to target antigens can be efficiently expressed not only in prokaryotic system but also in a plant expression platform, highlighting its versatility for scalable and cost-effective production.

## 4. Materials and Methods

### 4.1. Structure of Fusion Proteins

Gene encoding CP of phage PQ465 (GenBank PDB: 6YFS_AA) was synthesized in vitro and codon-optimized for expression in both *E. coli* and *N. benthamiana*. Superfolder variant of green fluorescent protein (GenBank MK995038) was used. To separate CP and GFP, we used flexible glycine-serine linker 19S (GTSGSSGSGSGGSGSGGGG) [50]. The proteins contained hexahistidine tags (6his) for purification of target products by metal affinity chromatography. The fusion proteins (Figure 1) contained the following elements: 6his-19S-GFP-19S-PQ465 (GFP-PQ465), PQ465-19S-GFP-6his (PQ465-GFP), and 6his-19S-GFP (GFP). The structures of monomeric proteins were predicted using Alphafold v2.3.1 [51] and visualized using SWISS MODEL [52]

### 4.2. Construction of Expression Vectors

Plasmids pQE30 and pQE60 (Qiagen, Hilden, Germany) were used to express recombinant proteins in *E. coli*. Previously obtained vector pQE30_19s-4M2e-19s-PQ465 [26], encoding N-terminal hexahistidine tag followed by four copies of M2e peptide of influenza A virus flanked by 19S linkers and CP of phage PQ465, was used to obtain N-terminal GFP fusion to the PQ465 capsid. The sequence encoding *gfp* gene was amplified by PCR using primers F_GFP_SacI (TATAGAGCTCATGCGTAAAGGCGAAGAGCT) and R_GFP_EcoRV (TATAGATATCTTTGTACAGTTCATCCATAC) and pWS082 plasmid (Addgene, Cambridge, MA, USA) as a template. The PCR fragment was cloned in pQE30_19s-4M2e-19s-PQ465 at the *Sac*I/*EcoR*V sites to replace 4M2e, resulting in the expression vector pQE30_6his-19s-GFP-19s-PQ465. The *BamH*I/*EcoR*V fragment containing the *gfp* gene was cloned into the pQE30 vector using *BamH*I/*Sma*I restriction sites resulting in the pQE30_6his-19s-GFP vector.

The plasmid encoding C-terminal GFP fusion to the PQ465 capsid was obtained in two steps. At the first stage, the 4M2e sequence in the vector pETM10_PQ465-19S-4M2eh [26] was replaced with the *gfp* gene, which was obtained using PCR with primers F_GFP_SacI (TATAGAGCTCATGCGTAAAGGCGAAGAGCT) and GFP_Xho_R (ATCTCGAGTTTGTACAGTTCATCCATAC). *Sac*I/*Xho*I restriction sites were used for cloning. At the second stage, the plasmid obtained in the first stage was used as a template for PCR with primers 6his_Hind_Sma_R (ATCCCGGGAAGCTTCGGATCTCAGTGGTGGTGGTGGTGGTGCTCGAG) and PQ465_apa_F (TAGGGCCCGCACAGCATAATATGCGTCT). The obtained fragment was cloned into the expression vector pQE60_PQ465 [26] at the *BamH*I/*Hind*III sites, resulting in the pQE60_PQ465-19S-GFP-6his vector.

For expression in plants, the *gfp-PQ465* fusion gene was amplified by PCR using the pQE30_6his-19s-GFP-19s-PQ465 plasmid as a template with primers: Pr-F PalI_his (ATATGGCGCGCCATGAGAGGATCGCATCAC) and PQ465_SmaI_R (ATCCCG GGTCAAGAACCTGTGTATTCTACAA). The *pq465-gfp* fusion gene was amplified by PCR using the pQE60_PQ465-19S-GFP-6his plasmid as a template with primers: PQ465_Asc_F (TAGGCGCGCCATGGCACAGCATAATATGCG) and 6his_Hind_Sma_R (ATCCCGGGAAGCTTCGGATCTCAGTGGTGGTGGTGGTGGTGC TCGAG). The obtained PCR fragments were cloned into the pEff expression vector at the *Asc*I/*Sma*I sites resulting in the pEff-GFP-PQ465 and pEff-PQ465-GFP vectors, respectively.

### 4.3. Expression of Recombinant Proteins in E. coli

Expression vectors were introduced into *E. coli* strain DLT1270, a derivative of DH10B, containing a *lacI* repressor gene integrated into the chromosome. Strains were grown at 37 °C in LB medium supplemented with 100 μg/mL ampicillin until mid-log growth phase (OD_600_~0.5). IPTG was then added to a final concentration of 0.5 mM, and the culture was grown overnight at 20 °C for pQE60_PQ465-19S-GFP-6his and at 30 °C for pQE30_6his-19s-GFP-19s-PQ465 and pQE30_6his-19s-GFP.

VLPs formed by PQ465-GFP and GFP-PQ465 proteins were purified by precipitation of the cell lysate with 25% saturated ammonium sulfate overnight at 4 °C. GFP was purified under non-denaturing conditions using metal-affinity chromatography on Ni-NTA-agarose (Qiagen). PQ465 CP was expressed and purified as described previously [26].

### 4.4. Expression of Recombinant Proteins in Plants

The pEff-GFP-PQ465 and pEff-PQ465-GFP expression vectors were introduced into *Agrobacterium tumefaciens* GV3101 by electroporation. The transformed agrobacteria were cultured in LB medium at 28 °C, supplemented with kanamycin (50 µg/mL), rifampicin (50 µg/mL), and gentamicin (25 µg/mL). The *A. tumefaciens* culture was centrifuged at 4000× *g* for 5 min to pellet the cells, which were then resuspended in an infiltration buffer containing 10 mM MES (pH 5.5) and 10 mM MgSO_4_, adjusted to an OD_600_ of 0.4. *N. benthamiana* plants were grown in a greenhouse in a 16 h daylight regime with additional illumination for about 6 weeks. The bacterial suspension was introduced into *N. benthamiana* leaves via needleless syringe infiltration, and leaf samples were collected 4 days post-infiltration. The leaves were ground in PBS buffer (50 mM sodium phosphate, 300 mM NaCl, pH 8.0), and the homogenate was passed through a paper filter and centrifuged at 14,000× *g* for 10 min to obtain the soluble protein fraction.

For purification of his-tagged proteins GFP-PQ465 and PQ465-GFP, the supernatant was mixed with Ni–NTA resin pre-incubated with PBS and incubated for 60 min with gentle agitation. The sorbent was then washed with PBS containing 10 mM imidazole and then 20 mM imidazole. Bound proteins were eluted with buffet containing 500 mM imidazole and subsequently dialyzed against PBS (1:100 ratio, four buffer exchanges) using a 3.5 kDa MWCO Slide-A-Lyzer Mini dialysis device (Thermo Fisher Scientific, Waltham, MA, USA).

### 4.5. SDS-PAGE and Western-Blotting of the Recombinant Proteins

The protein samples were mixed with half volume of the 3x sample loading buffer (40% glycerol, 4% SDS, 50 mM Tris pH 6.8, 1% bromophenol blue, 5% beta-mercaptoethanol). The obtained mixture was analyzed by SDS-PAGE or Western blotting. After SDS-PAGE, the gel was stained with Coomassie brilliant blue or was used to transfer proteins onto a Hybond-P membrane (GE Healthcare, Chicago, IL, USA) using the Trans-Blot Turbo Transfer System (Bio-Rad Laboratories, Hercules, CA, USA). Then the membrane was blocked with a 5% (*w*/*v*) solution of dry milk in TBS-T (20 mM Tris pH 8.0, 150 mM NaCI, 0.1% Tween 20) buffer for 1 h at room temperature. Subsequently, the membrane was incubated with primary antibodies for 1 h at room temperature. The following primary antibodies were used: mouse monoclonal anti-hexahistidine tag antibodies (HT501, TransGen Biotech, Beijing, China) at a 1:1000 dilution or rabbit monoclonal anti-GFP antibodies (2 mg/mL, used at 1:10,000 dilution). The membrane was washed 3 times for 15 min with TBS-T buffer and then incubated with anti-mouse secondary antibodies (IMTEK, Moscow, Russia) at 1:10,000 dilution or anti-rabbit secondary antibodies (Promega, Madison, WI, USA) at 1:10,000 dilution) conjugated with peroxidase for 1 h at room temperature. Then, the membrane was washed 3 times with TBS-T buffer (15 min at room temperature). Target protein-antibody complexes were detected using the Western Blot ECL Plus kit (GE Healthcare, New York, NY, USA) and the Fusion Solo X (Vilber, Eberhardzell, Germany) instrument.

### 4.6. VLP Assembly Analysis

The particle assembly was analyzed using a transmission electron microscopy (TEM) and dynamic light scattering. TEM was performed on a JEM 1400 microscope (JEOL, Tokyo, Japan). Protein samples were loaded on carbon-formvar-coated copper grids (TED PELLA, Redding, CA, USA) and stained with 1% (*w*/*v*) uranyl acetate in methanol. Dynamic light scattering analysis was performed using a Zetasizer NanoS90 analyzer (Malvern Instruments Ltd., Malvern, UK).

### 4.7. Analysis of Antigenic Properties of Recombinant Proteins Using ELISA

96-well microtiter plates were coated with serial dilutions of GFP-PQ465, PQ465-GFP, GFP and PQ465 proteins in sodium bicarbonate buffer pH 8.5. The proteins were applied to the membrane starting from a concentration of 1 μg per target protein, with subsequent dilutions. After washing with PBST (PBS with 0.05% Tween), the plates were incubated with blocking buffer (0.1% (*w*/*v*) BSA in PBS) for 1 h at 37 °C. After washing once with PBS, the plates were probed with rabbit monoclonal antibodies against GFP (2 mg/mL, used at 1:10,000 dilution) and mouse polyclonal antibodies against PQ465 (used at 1:2000 dilution). Peroxidase-labeled anti-mouse secondary antibodies (IMTEK, Russia) at 1:5,000 dilution or anti-rabbit secondary antibodies (Promega, USA) at 1:10,000 dilution were used a conjugate. Peroxidase activity was detected using tetramethylbenzidine substrate (Vector-BEST, Novosibirsk, Russia). The reaction was halted with 0.5 N HCI before measuring OD_450_ using a microplate spectrophotometer.

## 5. Conclusions

The CP of ssRNA phage PQ465 could be used as a carrier for presentation of relatively large antigens on the surface of recombinant VLPs, as exemplified by 238 a.a. long GFP. GFP was genetically linked to the N- or C-terminus of PQ465 CP, and the chimeric VLPs were efficiently produced in *E. coli* cells. GFP fusions did not interfere with VLP assembly. GFP was exposed on the surface of chimeric VLPs and limited the accessibility of the CP core for the antibodies. Chimeric VLPs, formed by CP containing GFP at the C-terminus were also efficiently expressed in *N. benthamiana* plants using self-replicating viral vector. Overall, the capsid protein of the bacteriophage PQ465 is a promising platform for constructing VLPs that present long peptide antigens on their surface, and such VLPs can be used in vaccine development studies.

## Figures and Tables

**Table 1:** Sizes of VLPs formed by PQ465-containing proteins produced in *E. coli*.

Method	PQ465-GFP	GFP-PQ465	PQ465
Dynamic light scattering ^1^	45.5 ± 5.5 nm	42.5 ± 3.9 nm	39.7 ± 4.7 nm
Transmission electron microscopy ^2^	34.3 ± 2.8 nm	34.9 ± 5.9 nm	28.8 ± 2.5 nm

^1^ Mean ± standard deviation (20 measurements). ^2^ Mean ± standard deviation (10 particles).

## Data Availability

The original contributions presented in this study are included in the article. Further inquiries can be directed to the corresponding author.
